# 妊娠期阵发性睡眠性血红蛋白尿症1例报告并文献复习

**DOI:** 10.3760/cma.j.cn121090-20240411-00137

**Published:** 2024-09

**Authors:** 蔚 王, 小钦 王

**Affiliations:** 复旦大学附属华山医院，上海 200040 Huashan Hospital Affiliated to Fudan University, Shanghai 200040, China

## Abstract

阵发性睡眠性血红蛋白尿症（PNH）为罕见病，妊娠期妇女的规范化治疗和随访管理对于孕产妇和新生儿的安危都非常重要。本文报告1例依库珠单抗治疗后顺利产子的妊娠期PNH患者，并对妊娠期PNH患者的治疗方案和随访管理进行总结，旨在提高孕产妇PNH的治疗水平。

## Abstract (en)

Paroxysmal nocturnal hemoglobinuria（PNH）is a rare disease, and a standardized treatment and follow-up management strategy for pregnant women with this disease are crucial for ensuring the safety of both the mothers and their newborns. This study includes a case of pregnant women with PNH. In accordance with the guidelines, the patient was successfully delivered after treatment with eculizumab. Furthermore, this study also implemented a treatment plan and follow-up management plan for PNH patients during pregnancy to elevate the standard of care for pregnant women with PNH.

阵发性睡眠性血红蛋白尿症（PNH）是一种造血干细胞突变引起的罕见的血液系统非恶性克隆性疾病。由于体细胞上PIG-A基因突变导致部分或完全血细胞膜糖化磷脂酰肌醇（phosphatidyl inositol, GPI）锚合成障碍，造成血细胞表面GPI锚连蛋白缺失，细胞灭活补体等能力减弱，从而引起细胞容易被破坏，发生溶血等。临床主要表现为不同程度的发作性血管内溶血、阵发性血红蛋白尿、骨髓造血功能衰竭和静脉血栓形成。依库珠单抗是一种C5补体抑制剂，可与补体蛋白C5特异性结合，从而抑制C5裂解为C5a和C5b，并防止末端补体复合物（MAC）的形成，从而减少血管内溶血，是指南推荐的首选治疗方案。现介绍一例我院诊治的妊娠期PNH患者，并结合该病例分享治疗体会。

## 病例资料

患者，女，33岁，2013年10月因“感冒后皮肤、巩膜发黄4 d，尿频伴血尿3 d”至当地医院就诊，血常规示：RBC 1.53×10^12^/L、HGB 53 g/L、WBC 5.8×10^9^/L、PLT 127×10^9^/L、网织红细胞比值（Ret％）19％；LDH：4 580 U/L；肝功能：总胆红素60.3 µmol/L、直接胆红素13.8 µmol/L、间接胆红素46.5 µmol/L；流式细胞术PNH检测：CD55^−^红细胞90.93％，CD55^−^粒细胞39.23％，CD59^−^红细胞94.45％，CD59^−^粒细胞39.35％。诊断为PNH。当时予输血、甲泼尼龙40 mg/d治疗。出院后口服甲泼尼龙24 mg/d，随后逐渐减量至8～12 mg/d维持，平时HGB在75～85 g/L，劳累、腹泻或感冒时可出现肉眼血尿，每年急性溶血发作1～5次，发作时HGB 50～70 g/L、Ret％ 10％～20％，LDH>2 000 U/L。2018年曾怀孕，但因为孕后期溶血急性发作，紧急剖宫产，胎儿死产。

本次孕20周入本院，流式细胞术检测示红细胞检出PNH克隆83.37％，粒细胞检出PNH克隆99.09％，单核细胞98.46％。完善脑膜炎疫苗接种后，于2023年9月27日第1次使用依库珠单抗600 mg静脉滴注，同时醋酸泼尼松15 mg/d口服，于10月8日、17日、25日行第2～4次依库珠单抗600 mg静脉滴注，11月2日按推荐方案行第5次依库珠单抗900 mg静脉滴注，以后每2周1次900 mg静脉滴注。于2024年1月10日剖宫产一健康男婴。术后仍规律用药，每2周1次依库珠单抗900 mg静脉滴注，产后患者HGB水平稳步上升至接近正常，婴儿未发现异常，治疗效果见表1。

**表1:** 妊娠阵发性睡眠性血红蛋白尿症患者依库珠单抗治疗情况及效果

日期	WBC （×10^9^/L）	HGB （g/L）	PLT （×10^9^/L）	Ret％	LDH （U/L）	备注
2023-9-25	8.25	85	94	7.58	1513	用药前
2023-9-27	–	–	–	–	–	第1次用药600 mg
2023-10-8	7.72	86	87	–	379	第1次600 mg后，第2次用药前
2023-10-17	7.32	87	83	8.91	308	第2次600 mg后，第3次用药前
2023-10-25	7.03	91	77	7.52	295	第3次600 mg后，第4次用药前
2023-11-2	6.83	90	74	9.82	302	第4次600 mg后，第5次用药前
2023-11-16	7.97	101	68	8.89	282	第5次900 mg后，第6次用药前
2023-11-30	7.34	98	62	–	324	第6次900 mg后，第7次用药前
2023-12-18	7.28	106	54	–	383	第7次900 mg后，第8次用药前
2024-1-2	6.24	94	57	–	395	第8次900 mg后，第9次用药前
2024-1-10	5.97	94	40	–	–	剖宫产一健康男婴（因为血小板减少至40×10^9^/L，提前2周剖宫产）
2024-1-17	5.70	93	74	5.83	447	第9次900 mg后，产后1周
2024-2-1	4.53	110	100	1.40	253	第10次900 mg后，产后3周

**注** Ret％：网织红细胞比值；–：无数据

## 讨论

PNH的发病率为1.59/100万～1.86/100万[1]。男女无明显差异，儿童发病率较少，发病高峰在30～45岁，所以患者中可见一定比例育龄期妇女。流行病学数据显示，PNH在亚洲（如日本、韩国和中国）比西方国家更常见[2]，但美国和欧洲的患者发生血栓栓塞的风险（30％～40％）高于亚洲患者（10％～15％）[3]。

PNH的主要症状包括贫血、血栓形成、平滑肌肌张力障碍，此外还有肾功能损伤、肺动脉高压、疲劳、腹痛和呼吸困难等。PNH在妊娠妇女中并发症更为常见，因为补体激活在妊娠期间增加，尤其是在孕20周后。

根据国际PNH工作组的分类，PNH分为3个主要类别：经典型PNH、合并其他骨髓衰竭性疾病（BMF）的PNH、亚临床型PNH[4]。目前经典型PNH则推荐依库珠单抗治疗，若疗效不佳，可考虑增加依库珠单抗的频率及剂量[5]。治疗剂量初始期为每周600 mg，持续4周；维持期为第5周开始900 mg，后续每2周900 mg。

2007年前，PNH患者的中位生存时间为15～20年[6]。在依库珠单抗问世后，患者的中位生存时间和普通健康人群已没有明显差别。目前一般建议在粒细胞PNH克隆>10％的患者中，实验室检查提示有明确的血管内溶血证据，并且有以下至少一项情况的，就应该开始依库珠单抗治疗：症状性贫血，尤其有输血依赖；血栓形成；肾功能不全；肺功能不全或肺动脉高压；腹痛至需要入院或阿片类镇痛剂；重度疲劳；妊娠。

在依库珠单抗应用之前，PNH母亲和胎儿的死亡率都很高[7]，母亲死亡率接近20％，主要是因为血栓栓塞事件，胎儿死亡率接近9％，主要是由于早产。依库珠单抗问世后，死亡率显著下降，根据2015年Kelly等[8]报道的在61例妇女中随访的共75次妊娠结果显示，没有孕妇死亡，胎儿死亡率为4％。依库珠单抗在孕妇中使用是安全的，脐血中浓度非常低，对胎儿补体系统无影响，母乳中未检测到[9]。在孕妇中使用依库珠单抗的研究较少，Kelly等[8]报道显示，使用依库珠单抗之后胎儿死亡率没有增加，活产的胎儿随访其视力、听力、运动能力、精细活动能力、行为、语言能力及身体状况均无异常，显示并无明显致畸生殖毒性。因此建议孕妇都应使用依库珠单抗治疗，并至少持续应用至分娩后3个月。由于孕后期补体激活增加容易发生突破性溶血，约有50％患者需要增加剂量（增至1 200 mg）或缩短给药间期（缩短至10～12 d）。本例患者于用药期间因血红蛋白水平稳定，未发生明显的突破性溶血，故原剂量治疗未增加剂量。血栓栓塞是PNH患者的主要死亡原因之一[10]，关于怀孕期间抗凝治疗的研究较少，但大多指南还是建议预防性抗凝，孕中期开始使用低分子量肝素，至少用到产后6周。

尽管依库珠单抗治疗有效，但它也可能会导致严重并发症，最常见的是危及生命的荚膜细菌感染，包括脑膜炎球菌感染。因此，所有患者均应提前2周接种脑膜炎球菌疫苗，若患者需紧急开始补体抑制治疗，应立即接种脑膜炎球菌疫苗并预防性使用抗生素2周。孕妇接种脑膜炎球菌疫苗是安全的。

对于初始应用依库珠单抗的患者，需评估治疗效果。随访内容应包括症状、体征、是否有输血需求、血常规、网织红细胞、LDH以及血管内溶血的各项指标。启动治疗的1个月内可以每周随访，以后每1～2个月检测1次。患者的LDH接近正常（即<1.5×正常参考值）认为溶血控制良好。此外，根据患者的情况随访相应指标，如对有栓塞病史或高危患者需随访D-二聚体，对有肺动脉高压的患者需随访脑利钠肽前体。如果出现可能使补体活化的状态，如感染、手术、怀孕等状况，应增加随访频率。对于有骨髓衰竭症的小克隆PNH患者，需随访流式细胞PNH克隆变化，一般6～12个月随访1次。对于孕妇，需要更频繁地随访，尤其在怀孕的前3个月，应该纳入高危产科的规范。

需要注意的是，接受依库珠单抗治疗的患者可能会发生血管外溶血[11]，血管外溶血的发生与C3补体激活有关，通常Coombs试验C3阳性，这种情况下患者的治疗与血管内溶血不同，可能需要使用糖皮质激素或脾切除，将来或可使用C3补体抑制剂治疗。本例患者并未发生血管外溶血。

依库珠单抗是第一个C5补体抑制剂，但是需要每2周1次长期静脉用药，针对这种情况，已经批准使用的瑞利珠单抗（ravulizumab）也是C5补体抑制剂，长效制剂，每8周使用1次，可伐利单抗（crovalimab）每4周使用1次，且可以皮下使用。除了C5补体抑制剂，还有针对其他靶点的抑制剂，如针对C3补体抑制剂pegcetacoplan也已在FDA获批使用，还有针对D因子的补体抑制剂和针对B因子的补体抑制剂均为口服制剂[12]，B因子抑制剂iptacopan也已经获批在国内上市，相信将来的PNH患者会有更多、更便利的治疗选择。

PNH是一种罕见的异质性疾病，对于孕产妇的危害较大。标准化的诊断和治疗规范是必须的，早期用C5补体抑制剂治疗，积极随访和处理各种并发症，有助于减少孕产妇和胎儿的死亡。本文我们成功治疗1例，依库珠单抗目前显示无明显致畸的生殖毒性，但是否对生育能力或在中国人群同样安全尚需更多病例评估药物安全性。
